# Post thrombolytic alveolar hemorrhage: a case report

**DOI:** 10.1093/omcr/omac145

**Published:** 2023-01-18

**Authors:** Mahmoud Mardenli, Mike Ghabally, Barkhodan Horo, Qais Alkhatib, Malek Raee

**Affiliations:** Department of Internal Medicine, University of Aleppo, Aleppo, Syria; Faculty of Medicine, Department of Internal Medicine, Division of Cardiology, Aleppo, Syria; Faculty of Medicine, Department of Internal Medicine, Division of Cardiology, Aleppo, Syria; Faculty of Medicine, Department of Internal Medicine, Division of Cardiology, Aleppo, Syria; Faculty of Medicine, Department of Internal Medicine, Division of Cardiology, Aleppo, Syria

## Abstract

Alveolar hemorrhage following thrombolytic agents administration is an extremely rare entity that has only been reported in twenty two patients in the medical literature. We herein report a case of a 60-year old male with an acute ST-elevation myocardial infarction who was treated with Streptokinase. Twelve hours after streptokinase adminstration, the patient developed severe hemoptysis and dyspnea and radiological studies were highly suggestive for acute alveolar hemorrhage. His past medical history is significant for severe chest trauma ten months prior to presentation. . Conservative therapy in addition to anti-coagulants withdrawal has led to gradual improvement in the next six days. We also discussed the aspects of our patient in comparison with published cases.

## INTRODUCTION

Alveolar hemorrhage (AH) is a life-threatening condition characterized by intra-alveolar bleeding. AH usually presents with anemia, hemoptysis, and dyspnea that can lead to respiratory distress syndrome and death [1–3].

Thrombolytic therapy is the mainstay of revascularization in patients with acute ST-elevation myocardial infarction (STEMI) who are not eligible for primary percutaneous coronary intervention (P-PCI) [1, 2]. However, it is associated with a non-negligible risk of bleeding involving the visceral organs such as gastrointestinal and genitourinary tracts, vascular access sites and intracranial hemorrhage [1].

AH following thrombolytic therapy is an extremely rare entity that has only been reported in 22 patients through the medical literature.

## CASE PRESENTATION

A 64-year old Caucasian male presented to our emergency department with a compliant of 6-hour retrosternal constricting chest pain with nausea and diaphoresis. Electrocardiogram showed a sinus rhythm of acute anterior STEMI. After appropriate loading of aspirin and clopidogrel, the patient was admitted to the coronary care unit and streptokinase 1.5million units was administrated. His chest pain resolved within three hours with minimal response to thrombolytic therapy on electrocardiogram. Unfortunately, rescue-PCI is not available in our country, thus the patient was continued on medical therapy.

His past medical history was significant for paroxysmal atrial fibrillation (treated with bisoprolol 5 mg) and a multiple trauma caused by a car accident ten months ago. His trauma caused seven rib fractures (four in the left, three in the right), pneumothorax, severe lung contusion, three vertebral fractures in the lateral process and hip fracture. He underwent thoracothentesis, vertebral fixation and total hip replacement and was discharged in a good condition with a mild pleural effusion on chest X-ray (CXR) and no pulmonary complaints.

Twelve hours after streptokinase administration, the patient experienced a severe acute hemoptysis, moderate dyspnea, respiratory rate elevation to thirty breath per minute, oxygen saturation drop (97%to 89%) and hemoglobin drop (12.8 mg/dL to 11.2 mg/dL) without any other source of bleeding. Laboratory tests were otherwise normal. He had no hyperthermia or other signs of pneumonia. Chest auscultation revealed bilateral basal to middle crackles without any other signs or symptoms of heart failure. Thus, the patient was initially treated with intravenous furosemide and increasing dose of nitrate without response.

Transthoracic echocardiography revealed hypokinesia in anterior wall and intraventricular septum with a 40% ejection fraction.

**Figure 1:**
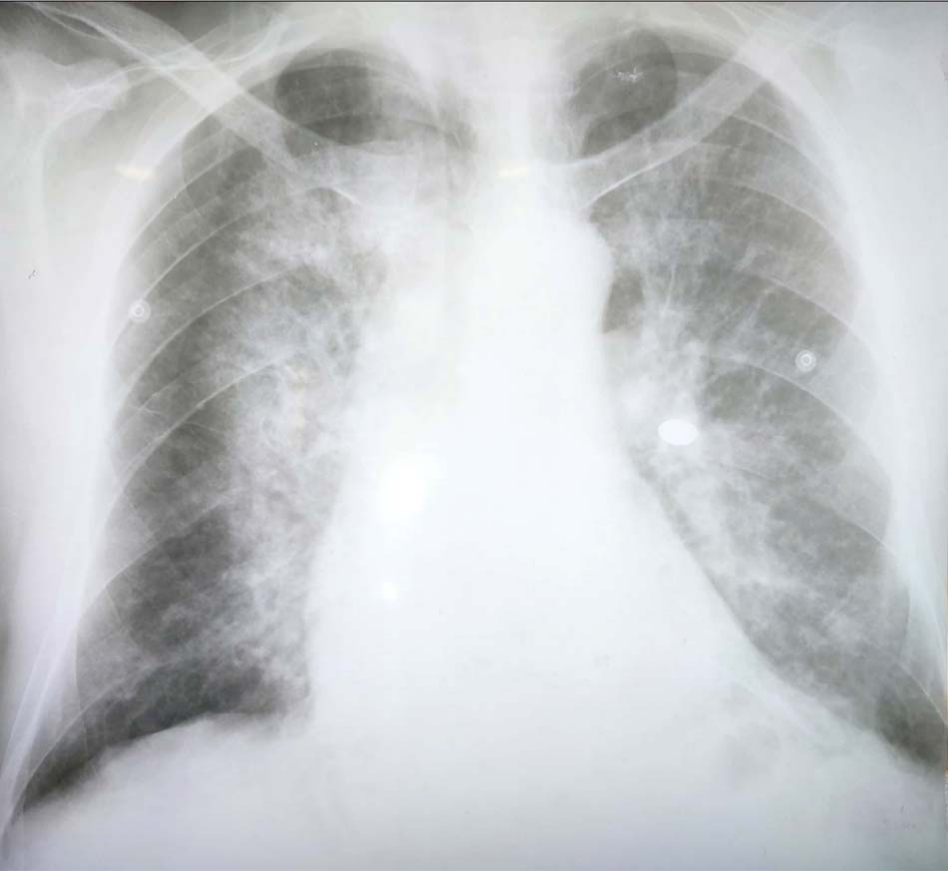
Bedside CXR. Bedside CXR demonstrates bilateral lung infilterates in the central portion of the lungs extending to the middle and peripheral area.

Bedside CXR demonstrated the presence of alveolar opacities involving the central portion of the lungs, particularly in the middle and lower lobes with mild pleural effusion, which directed us to the diagnosis of AH. [Figure 1].

**Figure 2:**
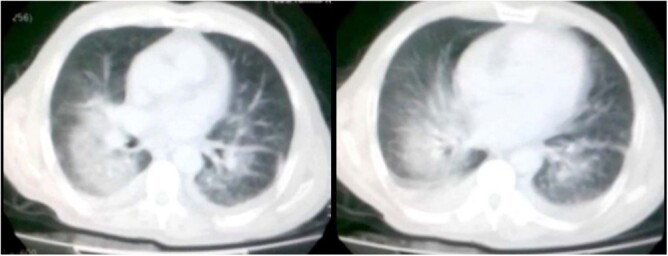
Chest Computerized Tomography. Chest computerized tomography demonstrates bilateral ground glass opacities in the central portion of the lung sparing into the bases; in addition to the presence of mild pleural effusion.

Chest computerized tomography scan demonstrated bilateral ground glass opacities in the central portion of the lung sparing into the bases. Which made the diagnosis of post-thrombolytic AH strongly suspected. [Figure 2].

Aspirin, clopidogrel and heparin sodium were withdrawn and oxygen was administrated continuously via nasal cannula. Later on, the patient experienced a significant improvement of dyspnea and hemoptysis within 48 hours, while hemoptysis did not completely resolved until the sixth day. On day six, CXR demonstrated normal lung fields. On day seven, the patient was discharged home on aspirin mono-antiplatelet therapy taking into consideration the bilateral nature, severe chest trauma, and thus a potential risk of recurrence.

Follow-up of the patient for sixty days was uneventful and coronary angiography revealed a 90% stenosis of the left anterior

descending artery, 80–90% long stenosis of the circumflex artery and 60% stenosis of the posterior descending artery.

## DISCUSSION AND CONCLUSION

Diffuse alveolar hemorrhage is a life-threatening condition characterized by a symptomatic triad of dyspnea, hemoptysis and anemia in addition to diffuse bilateral lung infiltrates on radiological studies [1, 3, 4]. The clinical course of the disease have a wide spectrum from asymptomatic mild cases to abrupt fetal cases of severe respiratory distress, hemodynamic instability and death [1, 3].

Thrombolytic therapy is the mainstay of revascularization of acute STEMI when P-PCI cannot be performed within 120 minutes [1, 2]. The main considerations of these agents remain the bleeding risk and allergic reactions. Bleeding is reported to occur in (0.2–5.9%) of patient; mainly from puncture sites, visceral cavities as gastrointestinal tract and genitourinary tract, retroperitoneum and intracranial hemorrhage [2, 5]. Unfortunately, the unavailability of P-PCI and other thrombolytic agents, makes streptokinase the only thrombolytic method in our country.

Chang YC et al [6] retrospectively reviewed 2634 patients with acute STEMI who received thrombolytic therapy and reported that hemoptysis has occurred in 11 patients (0.4%). However, frank AH is an extremely rare entity. Ben Murad et al review of the English medical literature revealed only 22 patients until date [1]. Surprisingly all 23 patients (including ours) were males, which posted the male gander as a predisposing factor [1]. Moreover, streptokinase was the thrombolytic agent in 16 out of 23 patients (69.5%). This can be contributed to the non-specificity of thrombolysis that is associated with an increased risk of bleeding in comparison with other specific fibrinolytic agents [1, 2]. Additionally, the immune reactions of streptokinase that varies from simple allergy to anaphylactic shock can be a contributing factor. It was suggested that immune-mediated capillaritis could be a possible etiology of AH following streptokinase administration [1, 2, 4]. According to Ben Murad review, only five fetal cases out of 23 cases were reported with a mortality rates of 21.7% [1].

Hammoudeh et al [5] reported a similar case of a unilateral AH in a patient with an ipsilateral chest trauma two years prior to the infarction. Taking into consideration that the trauma of our patient was bilateral. We believe that the severe trauma of our patient might have been a contributing factor of the AH. A possible etiology might be fibrinolysis state against any hemostating clot disassembling any bleeding from site of recent vascular injury [5].

The main differential diagnosis of post-thrombolytic AH is acute pulmonary edema due to either heart failure or mechanical complications, which can delay the diagnosis as in our patient. Differential diagnosis also includes thromboembolism, pneumonia and autoimmune disorders [1, 2].

Treatment options include both conservative management with respiratory support and blood transfusion; and causative management by withdrawal of all antiplatelet agents and anticoagulants, and, in severe cases the administration of tranexamic acid [1].

## DECLARATION

 

## ACKNOWLEDGEMENTS

None.

## CONFLICT OF INTEREST STATEMENT

No conflict of interest.

## FUNDING

There is no funding for this article.

## CONSENT FOR PUBLICATION

Informed written consent was obtained from the patient for publication of this report.

## AUTHORS’ CONTRIBUTIONS

Mardenli M was responsible for conception, critical revision of the manuscript for content and supervision.

Ghabally M was responsible for conception, design, acquisition, drafting and interpretation of data.

Horo B was responsible for critical revision of the manuscript, acquisition and design.

Alkhatib Q has contributed in acquisition, drafting and interpretation of data.

Raee M has contributed in acquisition, drafting and interpretation of data.

All authors reviewed and contributed to the final version of this case report. All authors read and approved the final manuscript.

## Supplementary Material

CARE-CHECKLIST_omac145
