# Supplementary material for: Post thrombolytic alveolar hemorrhage: a case report
Source: Oxf Med Case Reports. 2023 Jan 18;2023(1):omac145. doi: 10.1093/omcr/omac145 (PMC9853926; doi:10.1093/omcr/omac145)
Supplement: CARE-CHECKLIST_omac145 [file care-checklist_omac145.docx]

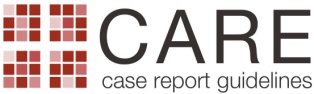
CARE Checklist of information to include when writing a case report
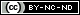


**Topic Item Checklist item description Reported on Line**

**Title 1** The diagnosis or intervention of primary focus followed by the words “case report” P1 Line 3

**Key Words 2** 2 to 5 key words that identify diagnoses or interventions in this case report, including "case report" P2 Line 32

Abstract

**(no references)**

**3a** Introduction: What is unique about this case and what does it add to the scientific literature? P2 Line 24

**3b** Main symptoms and/or important clinical findings P2 Line 25

**3c** The main diagnoses, therapeutic interventions, and outcomes P2 Line 28

**3d** Conclusion—What is the main “take-away” lesson(s) from this case? P2 Line 31

**Introduction 4** One or two paragraphs summarizing why this case is unique (**may include references**) P3 Line 33

**Patient Information 5a** De-identified patient specific information P3 Line 45

**5b** Primary concerns and symptoms of the patient P3 Line 46 and P4 Line 58

**5c** Medical, family, and psycho-social history including relevant genetic information P3 Line 52

**5d** Relevant past interventions with outcomes P3 Line 55

Clinical Findings

**Timeline**

**Diagnostic Assessment**

**Therapeutic Intervention**

**Follow-up and Outcomes**

1. Describe significant physical examination (PE) and important clinical findings P4 Line 58
2. Historical and current information from this episode of care organized as a timeline P4 Line 58

**8a** Diagnostic testing (such as PE, laboratory testing, imaging, surveys). P4 Line 58

**8b** Diagnostic challenges (such as access to testing, financial, or cultural) P4 Line 61, 66, 68 and 71

**8c** Diagnosis (including other diagnoses considered) P4 Line 73

**8d** Prognosis (such as staging in oncology) where applicable P4 Line 77

**9a** Types of therapeutic intervention (such as pharmacologic, surgical, preventive, self-care) P4 Line 74

**9b** Administration of therapeutic intervention (such as dosage, strength, duration) P4 Line 74

**9c** Changes in therapeutic intervention (with rationale) P4 Line 76

**10a** Clinician and patient-assessed outcomes (if available) P4 Line 76

**10b** Important follow-up diagnostic and other test results P5 Line 79

**10c** Intervention adherence and tolerability (How was this assessed?) P4 Line 76

**10d** Adverse and unanticipated events P4 Line 77

**Discussion 11a** A scientific discussion of the strengths AND limitations associated with this case report P5 Line 84

**11b** Discussion of the relevant medical literature **with references** P5 Line 84

**11c** The scientific rationale for any conclusions (including assessment of possible causes) P5 Line 96, P6 Line 113

**11d** The primary “take-away” lessons of this case report (without references) in a one paragraph conclusion P6 Line 122

**Patient Perspective 12** The patient should share their perspective in one to two paragraphs on the treatment(s) they received P8 Line 153

**Informed Consent 13** Did the patient give informed consent? Please provide if requested . . . . . . . . . . . . . . . . . . . . . . . . . . . . . . . . . . . . . . **Yes No**
